# A Secure IoT-Based Irrigation System for Precision Agriculture Using the Expeditious Cipher

**DOI:** 10.3390/s23042091

**Published:** 2023-02-13

**Authors:** Cherine Fathy, Hassan M. Ali

**Affiliations:** 1 Computer Engineering Department, College of Engineering and Technology, Arab Academy for Science and Technology (AAST), Alexandria 1029, Egypt; 2 National Training Academy, Sheikh Zayed City 12582, Egypt

**Keywords:** precision agriculture, lightweight encryption, MQTT, smart irrigation, IoT, expeditious cipher, AES, PRESENT, lightweight cryptography

## Abstract

Due to the recent advances in the domain of smart agriculture as a result of integrating traditional agriculture and the latest information technologies including the Internet of Things (IoT), cloud computing, and artificial intelligence (AI), there is an urgent need to address the information security-related issues and challenges in this field. In this article, we propose the integration of lightweight cryptography techniques into the IoT ecosystem for smart agriculture to meet the requirements of resource-constrained IoT devices. Moreover, we investigate the adoption of a lightweight encryption protocol, namely, the Expeditious Cipher (X-cipher), to create a secure channel between the sensing layer and the broker in the Message Queue Telemetry Transport (MQTT) protocol as well as a secure channel between the broker and its subscribers. Our case study focuses on smart irrigation systems, and the MQTT protocol is deployed as the application messaging protocol in these systems. Smart irrigation strives to decrease the misuse of natural resources by enhancing the efficiency of agricultural irrigation. This secure channel is utilized to eliminate the main security threat in precision agriculture by protecting sensors’ published data from eavesdropping and theft, as well as from unauthorized changes to sensitive data that can negatively impact crops’ development. In addition, the secure channel protects the irrigation decisions made by the data analytics (DA) entity regarding the irrigation time and the quantity of water that is returned to actuators from any alteration. Performance evaluation of our chosen lightweight encryption protocol revealed an improvement in terms of power consumption, execution time, and required memory usage when compared with the Advanced Encryption Standard (AES). Moreover, the selected lightweight encryption protocol outperforms the PRESENT lightweight encryption protocol in terms of throughput and memory usage.

## 1. Introduction

An increasing number of research studies on smart agriculture has been motivated by several challenges. First, the explosive growth of the world population [1]. According to the United Nations (UN) Food and Agriculture Organization, an increase of up to 70% more food will be required in 2050. Second, the declining agricultural lands and exhaustion of finite natural resources such as fresh water and arable land. In addition, the decreasing number of agricultural laborers in the majority of countries. As a consequence of this agricultural workforce decline, there is an urgent need for the adoption of IoT solutions in agriculture practices to reduce the need for manual labor. IoT solutions support farmers to tighten the supply-demand gap.

Precision agriculture integrates wireless sensor networks (WSNs) with traditional agriculture to improve crop yields. This is carried out by using a large number of low-power multi-function wireless communication sensors to remotely monitor the farmland to collect environmental data, crop growth data, and livestock health data to guarantee a reduction in the possible threats to the production process and help farmers make better decisions. Recently, there is a shift from the usage of WSNs for smart agriculture to the IoT as the main enabling technology for smart agriculture. The IoT combines several technologies such as radio frequency identification, wireless sensor networks, middleware systems, end-user applications, and cloud computing.

In [2,3], the authors presented an IoT ecosystem architecture for smart agriculture that is made up of four main components, namely: IoT devices, communication technology, internet, and data storage and processing. First, the IoT devices are responsible for monitoring the farming environment and collecting environmental data, crop growth data, and livestock health data. Second, the role of communication technology is to establish robust, reliable, and secure communication between the cloud and the farms. Wireless communication standards are categorized based on the coverage range into short-range and long-range standards. The short-range standards include Bluetooth, near-field communication (NFC)-enabled devices, ZigBee, Z-Wave, and passive and active radio frequency identification (RFID) systems. The long-range communication standards are defined as low-power wide-area networks (LPWA). Examples of long-range communication standards include LoRa, Sigfox, and NB-IoT. The LPWA technologies provide a wide area of coverage to low-power devices [4]. LPWA technologies outperform conventional cellular and short-range wireless technologies for different emerging smart city and machine-to-machine applications such as metering, logistics, industrial monitoring, and agriculture. However, LPWA technologies realize long-range ranging from a few to tens of kilometers and low-power operations at the expense of a low data rate. The primary aim of LPWA technologies is to achieve a 10-year battery life. LPWA technologies are suitable for delay-tolerant applications, as they achieve throughput in orders of ten kilobits per second and high latency in orders of seconds or minutes. Long range is achieved due to the use of the sub-1GHz band, and the deployment of narrow-band and spread spectrum techniques are the modulation techniques adopted by different LPWA technologies. In [4], the authors compared the technical specifications of various LPWA technologies and standards. The choice of communication technology depended on the application of the IoT device and the type of topology. Third, the internet is the core network layer enabling the availability of data collected by IoT devices anywhere and anytime. Routing Protocol for Low Power and Lossy Networks (RPL) [5] has been standardized by the Internet Engineering Task Force (IETF) as a routing protocol for resource-constrained nodes in IoT. RPL builds a robust topology over lossy links. A destination-oriented directed acyclic graph (DODAG) is the core of RPL, which represents a routing diagram of nodes. In [6], the authors proposed an enhanced routing protocol based on RPL called E-RPL that decreases the number of control messages. Moreover, they proposed a flexible multi-constrained objective function (OF) that integrates several metrics such as energy, delay, and bandwidth to define the end-to-end path between the sink and a given node. The simulation results of their proposal revealed a remarkable improvement in terms of end-to-end delay, energy consumption, and routing overhead. Finally, different platforms have been developed to provide data analytics, data management, and data storage of the big data collected from sensors. Data analytics (DA) has a primary role in improving the efficiency of smart agriculture systems and in increasing productivity. DA is classified into five classes based on the requirements of IoT applications: real-time analytics, memory-level analytics, offline analytics, business intelligence-level analytics, and massive analytics. In [7], the authors presented big IoT data analytic types, methods, and technologies for big data mining. DA can help in insurance, prediction, storage management, decision-making, farm management, and precision farming. In irrigation systems, the automated decision made by DA controls the water supply timing and quantity. The main objective of big data analytics is to analyze collected information to predict and identify recent trends, find hidden information, and finally, make decisions. Prediction, classification, clustering, and association rules are the main big data analytics methods.

Furthermore, smart irrigation is a precision agriculture application [8] that aims to control water consumption in the agriculture sector as a scarce resource in many countries by deploying IoT technology to remotely gather information from sensors implanted in agriculture terrains to monitor soil different parameters in all stages. Based on the collected information, a decision is made on when to irrigate and the water quantity and quality required. Table 1 reviews the recent research on IoT-based precision irrigation systems, highlighting the communication protocol, data analytics, and security techniques deployed in these systems. As can be noticed, recent research ignores security techniques and concentrates on the usage of machine learning and artificial intelligence approaches, such as fuzzy logic, artificial neural networks (ANNs), and regression models, to optimize IoT-based irrigation systems employing different environmental parameters and weather conditions to schedule the irrigation timing and the quantity of water used for irrigation. However, any alteration in the information coming from sensors and decisions passed to actuators can lead to crop damage, which is considered to be a crucial threat to the national security of any country. As such, a secure channel must be developed between the sensing layer and the decision-making entity to secure information flowing from the sensors to the decision-maker entity and to secure the decision returned to the actuators that control the irrigation system. Because precision agriculture is highly dependent on data and information from the monitored system, any alteration in such data during runtime can lead to expensive unmanageable decisions and actions from farmers. Therefore, there is a need to adopt the security mechanisms required to guarantee basic security functions: authenticity, reliability, integrity, and availability. Moreover, these security mechanisms must be lightweight to meet the requirements of constrained devices used in IoT.

In [1], the authors reviewed the previous work conducted on smart agriculture and highlights different aspects of applying IoT solutions in smart agriculture. Moreover, the article reviews smart agriculture’s related security issues and compares security issues in the industry (urban) and agriculture (rural).

In [16], the authors presented a classification of security threats in smart agriculture and precision agriculture environments. The authors classified security threats into six possible attacks: attacks on hardware (side channel attack and radio frequency (RF) jamming), attacks on the network equipment (denial of service (DoS), MITM (man in the middle), botnets, cloud computing attacks), attacks on data (data leakage, ransomware, cloud data leakage, false data injection, misconfiguration), attacks on applications (software update attacks, malware injection, buffer overflow, indirect attacks (SQL injection)), attacks on support chain (third-party attacks, data fabrication), and misuse attacks (cyber-terrorism, invalidation, and compliance). Another classification of security threats is underlined in [17]. The authors classified the security requirements in smart agriculture into six challenges, namely: integrity, availability, authentication, confidentiality, privacy, and Non-repudiation, and highlighted the possible attacks under each challenge. Moreover, a review of existing solutions to IoT security problems is emphasized. In [8], the authors added data freshness, authorization, and self-healing to the security requirements of smart agriculture. In [18], the authors reviewed IoT communication technologies security aspects for smart agriculture. In [19], the authors reviewed all categories of security attacks and the application of WSNs in IoT along with an evaluation of the countermeasures adopted against each type of attack.

Moreover, smart irrigation systems developed based on IoT technology consist of constrained nodes in terms of power, memory, and processing resources. As a result, conventional security protocols can not be supported in such systems [20,21]. Transport layer security (TLS) adds overhead in terms of memory and energy on constrained nodes. As a result of the dependence on constrained nodes in IoT-based irrigation systems, a lightweight security protocol must be deployed. Lightweight cryptography techniques balance throughput against power drain, memory usage, and gate equivalent and have lower performance when compared to cryptography standards (such as AES and SHA-256) [22]. Characteristics of lightweight cryptography are highlighted in ISO/IEC 29192 and ISO/IEC JTC 1/SC 27. Lightweight properties are evaluated based on chip size and energy consumption and small code and/or RAM size in case of software implementation [21]. In [21], the authors discussed privacy in IoT in the context of developing solutions and frameworks that address profiling and tracking, localization, and tracking challenges and underlined state-of-the-art lightweight cryptographic framework for IoT. In [23], the authors proposed a set of lightweight security protocols for encryption, authentication, and key management for IoT. The authors compared their proposed protocols with IPsec in terms of security and computational efficiency. They succeeded in achieving a decreased level of resource consumption with an increased level of security. In [24], the authors implemented AES and PRESENT ciphers on a smartphone and provided a performance evaluation comparison between the two algorithms. AES is the symmetric block cipher defined by the National Institute of Standards and Technology (NIST) as the standard for bulk data encryption, whereas PRESENT is a symmetric ultra-lightweight block cipher that was standardized by ISO/IEC.

**Figure 1:**
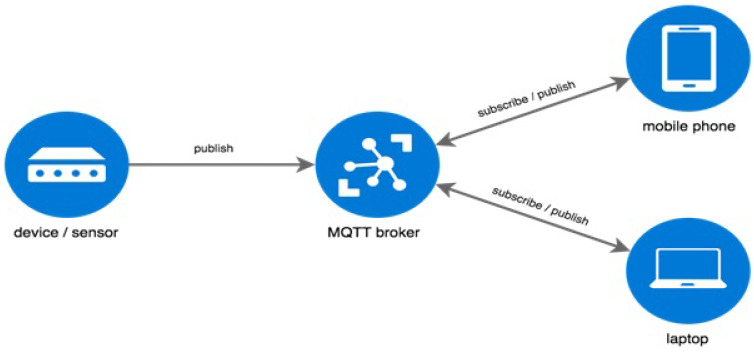
MQTT architecture.

On the other hand, the Message Queue Telemetry Transport (MQTT) protocol has been widely deployed as an application layer messaging and information exchange protocol in machine-to-machine (M2M) communication [25]. This is due to its ability to function with resource-constrained devices that utilize low bandwidth and unreliable links. MQTT is considered a lightweight, energy-efficient, and bandwidth-efficient communication protocol. MQTT utilizes the publish/subscribe architecture model to provide transition flexibility and simplicity of implementation. MQTT’s main components are the publishers (lightweight sensors), the subscribers (applications interested in sensor data), and the brokers (connect publishers and subscribers and classify sensor data into topics) as illustrated in Figure 1. The data generated by a publisher are dispatched to multiple subscribers through an MQTT broker. MQTT was proposed in 1999 by Andy Stanford-Clark of IBM and Arlen Nipper of Arcom and is currently an OASIS (Organization for the Advancement of Structured Information Standards) standard; it also has a standard defined in ISO/IEC 20922: 2016.

Motivated by the increasing importance of smart irrigation systems in conserving water as a scarce natural resource, the role of precision agriculture in agriculture development, and the urgent need to apply information security techniques in the IoT part of the smart agriculture ecosystem, our aim in this research is to integrate a lightweight cryptography layer into the IoT ecosystem for smart agriculture and to investigate the deployment of a lightweight encryption protocol (the Expeditious Cipher) to create a secure channel between the sensing layer and the broker of MQTT protocol as well as between the broker and its subscribers in smart irrigation systems. This secure channel protects the sensors’ published sensitive data from eavesdropping and theft and preserves the integrity of data, in addition to protection of the decision that is made by the DA entity and returned to actuators. It should be noted that the security in IoT-based systems lies in IoT local systems consisting of devices constrained in energy and computing power.

The following points summarize the main contributions of this article.

1. The main contribution of this article is the integration of a lightweight cryptography layer to the IoT ecosystem for smart agriculture that meets the requirements of constrained devices used in smart agriculture in general and specifically, in our proposed IoT-based irrigation system.
2. The article investigates the deployment of a lightweight encryption protocol (Expeditious Cipher (X-cipher)) to create a secure channel between the sensing layer and the broker in the MQTT protocol as well as a secure channel between the broker and its subscribers in smart irrigation systems (our case study).
3. The proposed model is evaluated through simulation to validate the lightweight property of the chosen encryption protocol in terms of power consumption, execution time, and memory usage. Moreover, a performance comparison is carried out between the Expeditious Cipher (X-cipher), AES, and PRESENT cipher (lightweight standard protocol) in terms of power consumption, execution time, memory usage, and average throughput.
4. The security requirements of IoT-based agriculture systems and the potential attacks against them are discussed.
5. A state-of-the-art lightweight security architectures proposed for securing MQTT protocol is reviewed after highlighting the concept of lightweight cryptography.

The remainder of the article is organized as follows: Section 2 presents our proposed secure smart irrigation system after briefly reviewing the state-of-the-art proposed lightweight security architectures for securing MQTT protocol. Section 3 highlights and discusses the performance evaluation results of our selected lightweight encryption algorithm versus AES, in addition to a performance comparison between X-cipher and the PRESENT cipher. Section 4 summarizes the findings of the article and highlights our suggestions for future work.

## 2. Materials and Methods

In this section, the proposed secure IoT-based irrigation system architecture is outlined. First, a brief review of the proposed lightweight security techniques for securing the MQTT protocol is given. Then, the proposed model architecture and hardware components are explained in detail. In addition, the applicability of the selected lightweight protocol (X-Cipher) on NodeMCU is verified.

### 2.1. MQTT Security Challenges and Solutions

MQTT runs over TCP with few security techniques such as simple authorization policies and basic authentication techniques. TLS/SSL with session key management has been suggested for securing MQTT; however, proposed security techniques for MQTT should be lightweight for deployment in IoT environments. TLS/SSL has been proven to have performance issues in terms of processing time, memory required, and energy consumption, in addition to the various possible attacks such as Heartbleed, CRIME, BEAST, and RC4. Furthermore, TLS does not provide fine-grained access control [26]. Moreover, MQTT suffers from several security shortcomings, mainly from a lack of confidentiality, integrity, availability (DOS attack), mutual authentication, access control, control message security, and end-to-end security. In [27], the authors reviewed the symmetric, asymmetric, and hybrid lightweight schemes proposed in the literature for guaranteeing the confidentiality of transmitted data using MQTT. Moreover, the authors’ review suggested security techniques for guaranteeing access control in MQTT. According to [28], the security threats against MQTT are mainly replay attacks and man-in-the-middle attacks (MITM). Value-to-keyed-hash message authentication code (Value-to-HMAC) has been deployed to achieve information confidentiality. In [26], the authors designed a security architecture for MQTT-SN to achieve end-to-end security in addition to fine-grained access control. Moreover, they introduced a certificate subject to create a secure direct channel between a publisher and a set of subscribers. In addition, they designed security schemes to integrate mutual authentication and control message security functions into the standard MQTT-SN control messages, without relying on the TLS. In [20], the authors proposed ChaCha20-Poly1305 Authenticated Encryption with Associated Data (AEAD) as a solution to secure resource-constrained node communication over MQTT/MQTT-SN. RFC 6574 [29] describes constrained nodes as they are bandwidth constrained, energy constrained, and memory constrained. Performance evaluation of the proposed scheme revealed a low memory requirement and low processing time. In [30], the authors presented a lightweight authentication and encryption mechanism based on ECDHE-PSK (Elliptic Curve Diffie–Hellman Ephemeral)-(Pre-Shared Key) for IoT-constrained devices. The proposed security mechanism outperforms the ECDHE-ECDSA in all performance evaluation tests.

### 2.2. Proposed IoT System Architecture

**Figure 2:**
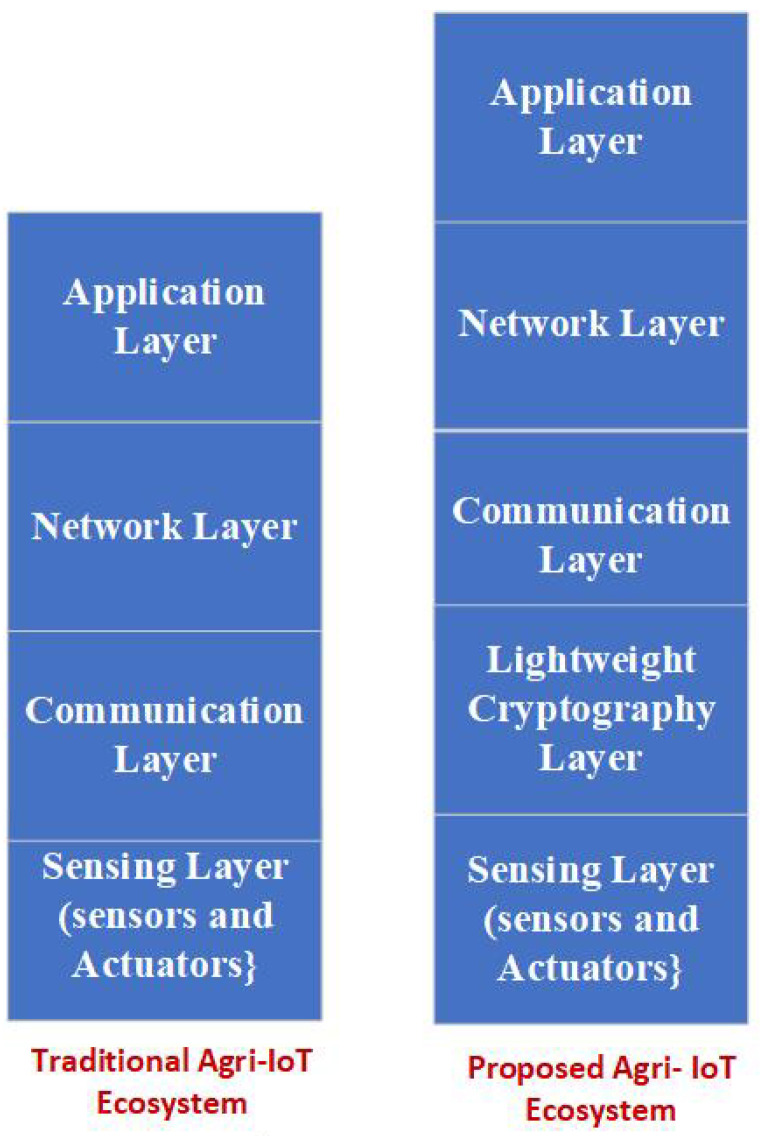
Traditional Agri-IoT ecosystem versus proposed Agri-IoT ecosystem.

The main objective of our proposed system is the addition of a lightweight cryptography layer to the agriculture IoT ecosystem, as shown in Figure 2. The function of this layer is to create a secure communication channel between the sensing/actuator layer (IoT nodes) and the subscriber layer, thus protecting sensors’ published data from eavesdropping and theft, as well as from unauthorized changes to sensitive data that can negatively impact crop development. In addition, the secure channel protects the irrigation decision made by the data analytics (DA) entity regarding the irrigation time and the quantity of water that is returned to actuators from any alteration while meeting the requirements of IoT-constrained devices in terms of memory usage and power consumption by ensuring the application of lightweight cryptography techniques.

**Figure 3:**
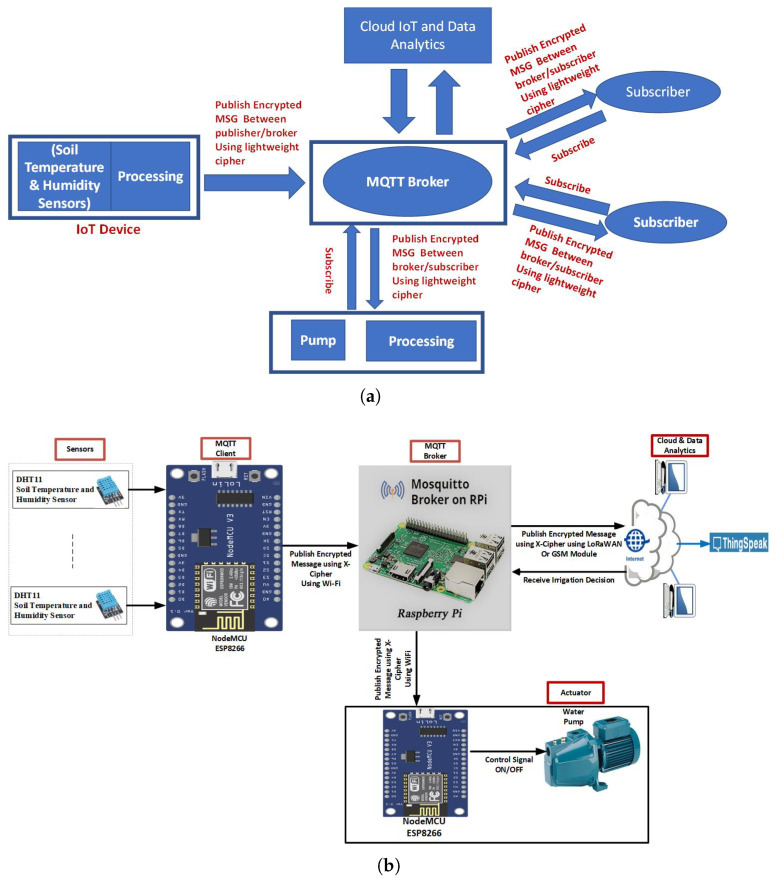
The proposed model. (**a**) proposed model architecture. (**b**) proposed model hardware.

Figure 3 depicts the architecture of our proposed secure IoT-based smart irrigation system. Figure 3a illustrates the general system architecture and Figure 3b shows the hardware components of the proposed system. The proposed architecture consists of five layers: the sensing/actuator layer, the lightweight cryptography layer, the communication layer, the network layer, and the application layer.

#### 2.2.1. The Sensing Layer

The sensing layer is made up of multiple low-power sensors that are spread on agricultural land to continuously collect soil temperature and humidity. The DHT11 sensors are used to measure the temperature value in degrees Celsius and the humidity value in percentage. The DHT-11 sensor has three pins: two of which are for power and one is for output data transmission. The three pins are used to connect the DHT11 sensor to the NodeMCU ESP8266 board. The DHT-11 library must be added to the Arduino IDE software before uploading to the NodeMCU ESP8266 board the code written to read the humidity and temperature value from the DHT11 sensor. Together, the DHT-11 sensor and the NodeMCU form the IoT node. The NodeMCU runs the MQTT client code and publishes the sensed soil parameters (the temperature and humidity readings) to the MQTT broker after encrypting the message using the Expeditious Cipher (X-cipher). The MQTT broker runs on Raspberry Pi, which is a low-power single-board computer. NodeMCU ESP8266 has a built-in WiFi module that is used as the communication protocol between NodeMCU and the Raspberry Pi. The MQTT protocol is chosen due to its short message transmission capability and low-bandwidth requirement, which makes it more convenient for machine-to-machine (M2M) communication. Moreover, the Raspberry Pi was selected as a processing layer because of its low cost and common use in IoT applications. Mosquitto is an open-source lightweight MQTT broker and is selected as it is suitable for Raspberry Pi. The Mosquitto broker decrypts the message and adds the information in the message to the related topic. Then, information is encrypted again before being published (broadcast) to all subscribers. The ThingSpeak cloud platform is used among subscribers that execute cloud analysis for making irrigation decisions regarding the time of irrigation and the quantity of water. Then the irrigation decision is published to the broker to be decrypted and then encrypted again before being published to the actuator. The nodeMCU at the actuator layer decrypts the message and then passes the control signal to the attached pump to activate the irrigation.

#### 2.2.2. Lightweight Cryptography Layer

In this layer, a lightweight protocol must be deployed, because the proposed IoT-based irrigation system is made up of constrained devices in terms of memory and energy. Characteristics of lightweight cryptography were highlighted in ISO/IEC 29192 and ISO/IEC JTC 1/SC 27. Lightweight properties are evaluated based on chip size and energy consumption, and small code and/or RAM size in case of software implementation [21]. So, the Expeditious Cipher (X-cipher) was selected [31]. The X-cipher was proposed in 2011 and is considered a lightweight high-throughput encryption protocol. The cipher sub-keys are generated using the well-studied SHA-512 and Whirlpool 512 hash functions. The two hash functions are cascaded in a pseudo-random manner that depends on the user key to enhance the cipher security. The cipher utilizes a variable-size user key. The encryption rate can attain 512 bits per cycle in the case of hardware implementation with parallelization encryption paths. Eight rounds are recommended for the proper operation of the Expeditious Cipher (X-cipher). The circuitry of the encryptor and decryptor are identical, as such, a high code density and small implementation area are achieved, as required by lightweight cryptography. An additional important feature of the X-cipher is the separation of key scheduling process from the encryption process, which allows the change of cipher design, key, and block size by simply changing the hash function. The Expeditious Cipher (X-cipher) complete encryption algorithm is presented in [31]. NodeMCU has a cryptography module called crypto that contains various functions for working with cryptographic algorithms. The AES 128-bit is supported in ECB and CBC modes, in addition to several hash functions, namely, MD5, SHA-1, SHA-256, SHA-384, and SHA-512. The MQTT module implemented in C language is available on the NodeMCU ESP8266 board to run the MQTT client to publish the temperature and humidity readings to the MQTT broker running on Raspberry Pi. So, because the proposed Expeditious Cipher (X-cipher) depends on the SHA-512 hash function that is already implemented in the crypto module and the modulo 2 addition between the hashed key and the plain text, the selected lightweight algorithm can be compiled easily on the NodeMCU board. Table 2 compares the operational features of AES (traditional encryption protocol), PRESENT (lightweight standard protocol), X-cipher (selected lightweight protocol), and the lightweight encryption protocol proposed recently in the literature.

#### 2.2.3. Communication Layer

The communication between NodeMCU ESP8266 and the Raspberry Pi is achieved using WiFi, as the NodeMCU has a built-in WiFi module. On the other hand, the communication between the Raspberry Pi and the internet can be carried out using LoRaWAN technology taking into consideration the low data rate characteristic of LPWA technologies discussed in Section 1 or through the GSM module.

## 3. Results

In this section, we validate through simulation that the chosen lightweight cipher satisfies the requirements of constrained devices in IoT-based smart irrigation systems in terms of power consumption and memory usage. Moreover, we compare the chosen lightweight cipher with PRESENT, a standard lightweight protocol, and AES, a standard encryption protocol.

### 3.1. Simulation Scenario

**Figure 4:**
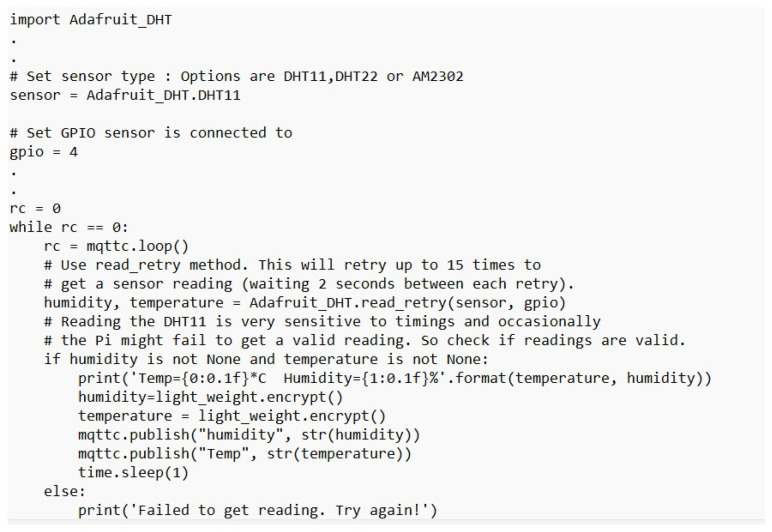
The Raspberry Pi emulator code for obtaining humidity and temperature readings from sensors.

**Figure 5:**
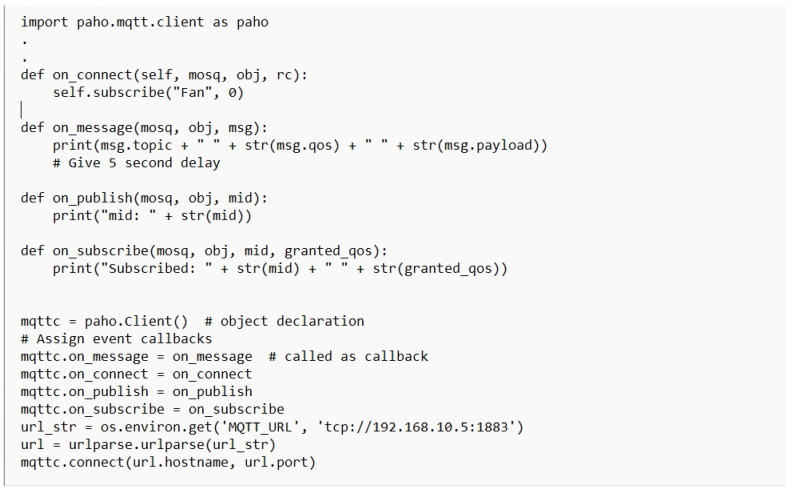
MQTT client code for the Raspberry Pi emulator.

**Figure 6:**
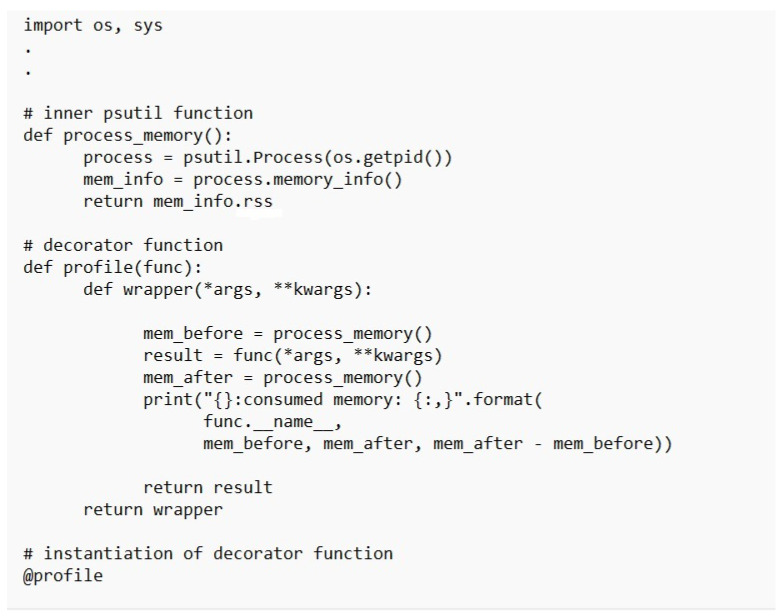
Memory usage calculation function.

**Figure 7:**
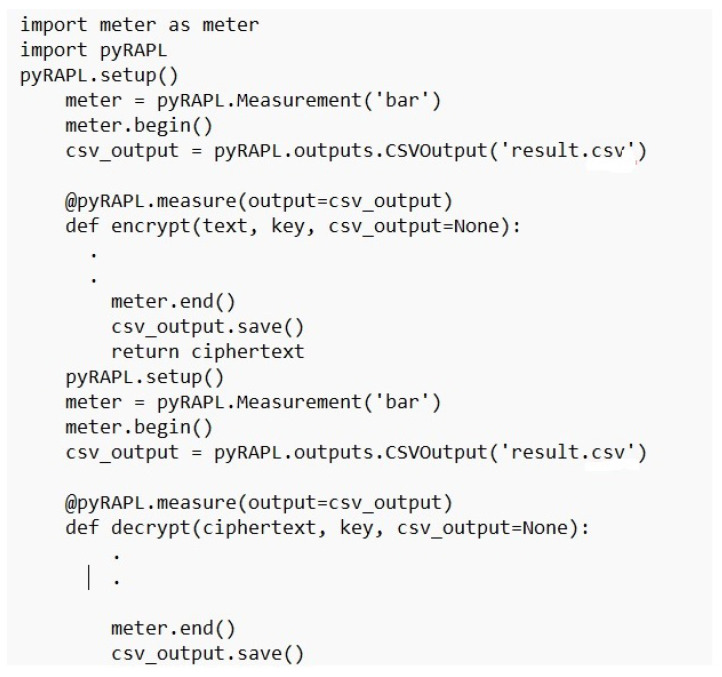
Power consumption calculation function.

Table 3 presents the simulation packages used in the performance evaluation of the X-cipher. A Windows 10 virtual machine runs the Raspberry Pi emulator that was selected. An Ubuntu 20.1 virtual machine is used for the subscriber. The DHT-11 sensors are connected directly to the Raspberry Pi Emulator. Figure 4 illustrates selected parts of the Python code used to obtain humidity and temperature reading from DHTT-11 sensors. The Adafruit_DHT Python library is available for reading the DHT series of humidity and temperature sensors on a Raspberry Pi. First, the sensor type and the pin that the DHT-11 is connected through to Raspberry Pi must be specified. Then, the function Adafruit_DHT.read_retry is used to read from the specified pin. As can be noticed, the humidity and temperature readings are encrypted using the lightweight class function encrypt, which is our class implementation for the X-cipher protocol. Moreover, Figure 5 depicts a selected part of the MQTT client code for the Raspberry Pi emulator. The paho.mqtt.client Python library is deployed to provide a client class with support for MQTT protocol, and it also provides some helper functions that facilitate publishing messages to an MQTT server. Figure 6 highlights selected parts of the python code used to calculate memory usage. Calculations of memory usage rely on the process.memory_info and choosing the “rss” option returns the actual physical memory the process is using. Figure 7 illustrated selected parts of the Python code used to calculate power consumption. The pyRAPL Python library is utilized to measure the energy footprint of a host machine along with the execution of a piece of Python code. Three performance metrics were used to evaluate and compare the Expeditious Cipher (X-cipher) and the AES cipher [32]: power consumption, execution time, and memory usage for a file of 10,000 message comma separated of total size 20 MB.

**Figure 8:**
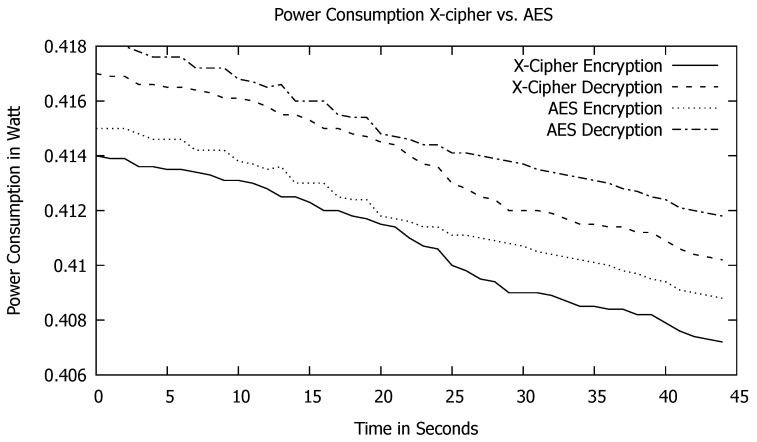
Power Consumption during encryption and decryption processes for AES and X-cipher.

Figure 8 compares the two algorithms’ power consumption during the encryption and decryption process. As shown in the graph, X-cipher requires less power consumption than AES throughout the whole encryption and decryption process. This makes X-cipher more convenient for IoT devices with limited energy resources. This is due to the dependency of X-cipher on just the XOR function between the generated hashed sub-key and the plain text, whereas AES depends on substitution–permutation operations. Moreover, the number of rounds in X-cipher is just eight, whereas the number of rounds is ten in the case of AES.

**Figure 9:**
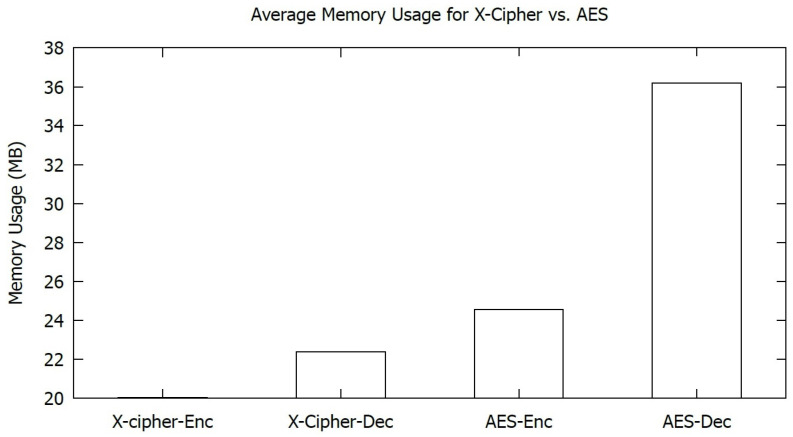
Average Memory usage for AES and X-cipher.

Figure 9 compares the memory usage during the encryption and decryption process for the two algorithms. As shown in the graph, X-cipher requires less memory than AES throughout the whole encryption and decryption process. X-cipher has average memory usages of 20.04 MB and 22.38 MB during the encryption and decryption processes, respectively, in contrast to AES, which has average memory usages of 24.56 MB and 36.2 MB during the encryption and decryption processes, respectively. The higher memory usage in the case of AES is due to the substitution–permutation principle employed in AES. This implies that X-cipher is more suitable for IoT devices with constrained memory capacity.

**Figure 10:**
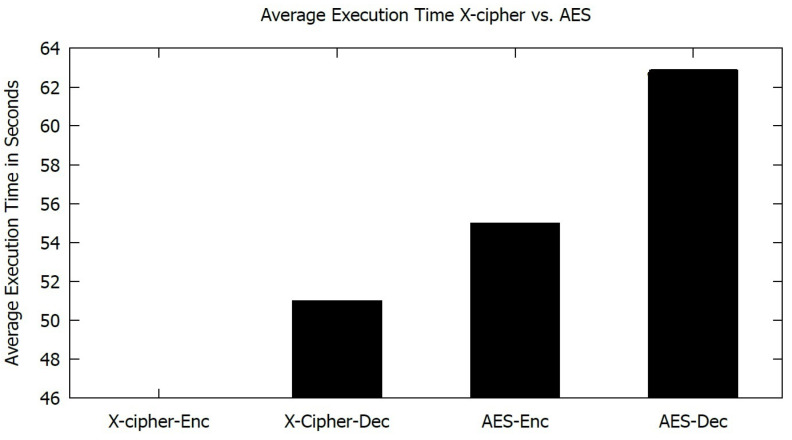
Average Execution time for AES and X-cipher.

Figure 9 presents the average memory usage for both algorithms for the encryption and decryption of 20 MB messages. Figure 10 illustrates the average execution time for AES and X-cipher. As shown in both figures, the selected lightweight algorithm (X-cipher) outperforms AES in terms of average memory usage and average execution time.

### 3.2. Comparison with Related Work

In this section, we compare the performance of the X-cipher versus the PRESENT ultra-lightweight cipher [33] presented in the literature for the same size of data of 20 MB. Our selected algorithm X-cipher outperforms the PRESENT algorithm in terms of throughput and memory usage as shown in Table 4 and Table 5. This is because PRESENT deploys the substitution permutation network principle although it has a reduced substitution box and due to the higher number of rounds, which is 31 rounds.

## 4. Conclusions

Smart irrigation systems integrate IoT technology with smart agriculture to conserve water consumption during the irrigation of agricultural land. This research focuses on the evaluation of the adoption of a lightweight security protocol to secure communication in smart irrigation systems. The adopted encryption algorithm (Expeditious Cipher) creates a secure channel between publishers and the broker of the MQTT protocol as well as between the broker and its subscribers. MQTT was chosen as the IoT application protocol for messaging in our proposed irrigation system, as it is a bandwidth- and energy-efficient protocol. The evaluated lightweight protocol reduces resource consumption in terms of memory usage and power consumption as well as minimizes the execution time, which makes it suitable for IoT-based resource-constrained systems. There is a trade-off between the security level and the memory and energy-processing resources required. AES achieves a higher security level at the cost of more memory usage and energy consumption, which causes performance degradation in constrained devices, opposite to lightweight cipher, which achieves an adequate level of security with low memory usage and reduced energy consumption. Comparing our selected X-cipher protocol with the PRESENT cipher reveals a lower memory usage and higher throughput in favor of the X-cipher. In the future, we will investigate the integration of our proposed system with software-defined networking (SDN) technology to implement the security models at the controller to offload publishers and brokers from the additional processing required by security algorithms. Moreover, we will develop a deep-learning model to predict the irrigation time and the required amount of water required for irrigation.

## Figures and Tables

**Table 1:** Literature review of recently proposed IoT-based precision irrigation systems.

Proposed Model	Security Techniques Used?	Communication Technology Deployed	Data Analytics (DA) Techniques Used ?	Challenge
Machine Learning-based Irrigation System (2019) [9]	Not used	LoRa P2P	Multiple linear regression algorithm used to calculate the amount of water required for each irrigation	Lack of security techniques
The AREThOU5A IoT Platform (2021) [10]	Yes, TCP/IP SSL for encryption	LoRaWAN	Not used	The research lacks evaluation of the proposed security techniques and does not apply lightweight security algorithms as TLS techniques add overhead in terms of memory and energy on constrained nodes
Deep Learning Intelligent Irrigation System for Agriculture (DLISA) (2021) [11]	Not used	Wireless communication but not specified	LSTM RNN model is proposed to predict the volumetric soil moisture of the next day to schedule irrigation	Lack of security techniques
Sensor-based Smart Irrigation System (2021) [12]	Not used	WiFi	ThingsSpeak displays graphs of collected soil parameters, irrigation decision is made by farmers to activate/deactivate the irrigation	Lack of security techniques
Secure Multi-Crop Smart Irrigation System (SMCSIS) (2021) [13]	Yes, access control techniques & Blockchain technology for privacy and data integrity	Not specified	A supervised feed-forward neural network (FFNN) to estimate evaporation for estimated soil moisture (ESM) calculation to specify the next irrigation time.	The research lacks evaluation of the proposed security techniques
Machine learning-based Irrigation System (2022) [14]	Not used	Lora	Support vector machine (SVM) and k-nearest neighbor (KNN) algorithms used for prediction of irrigation scheduling	Lack of security techniques
Intelligent Sensor-based Smart Irrigation System (2023) [15]	Not used	Lora	Recurrent neural network to forecast the soil moisture level	Lack of security techniques
Our proposed model Secure IoT-based Irrigation System (2023)	Yes, The Expeditious Cipher (X-cipher)	WiFi and LoRAWAN or GSM	ThingsSpeak displays graphs of collected soil parameters	The proposed model integrates lightweight cryptography to the IoT ecosystem of smart irrigation systems

**Table 2:** Comparison between X-cipher, AES, PRESENT, and lightweight encryption.

Protocol	Type	Key	Throughput	Number of Rounds	Comment
AES [32]	Symmetric block cipher	Different key for each round	128 bits per round	10	Security depends on bit permutation and S-box
X-cipher [31]	Symmetric block cipher	Different key for each round	512 bits per round	8	Security depends on cascaded hashed functions (SHA-512 and Whirlpool-512) to generate cipher sub- keys
PRESENT [33]	Symmetric block cipher	Different key for each round	64 bits per round	31	Security depends on bit permutation and S-box
Lightweight encryption [23]	Symmetric block cipher	Different key for each file	Not specified	Not specified	Security depends on secure key management protocol

**Table 3:** Software versions deployed in performance evaluation.

Software	Description
Python 2.7.18	Programming language
Raspberry Pi emulator 4.19.0-13-amd64	Raspberry Pi emulator
Ubuntu 20.1	Virtual machine
Windows 10	Virtual machine
Mosquito	Open source MQTT broker
Raspberry Pi OS with desktop Debian version: 11 (bullseye)	Raspberry Pi operating systems

**Table 4:** Throughput results comparison between X-cipher and PRESENT.

Protocol-Process	Throughtput KBps
X-cipher-encryption	164 KBps
X-cipher-decryption	172 KBps
PRESENT-encryption	140 KBps
PRESENT-decryption	140 KBps
AES-encryption	200 KBps
AES-decryption	210 KBps

**Table 5:** Memory usage results in comparison between X-cipher and PRESENT.

Protocol-Process	Memory Usage (MB)
X-cipher-encryption	20.04 MB
X-cipher-decryption	22.38 MB
PRESENT-encryption	90 MB
PRESENT-decryption	105 MB

## Data Availability

Not applicable.

## Abbreviations

The following abbreviations are used in this manuscript:

AEAD	Authenticated Encryption with Associated Data
AES	Advanced Encryption System
AI	Artificial Intelligence
ANNs	Artificial Neural Networks
DA	Data Analytics
DOS	Denial Of Service
DODAG	Destination-Oriented Directed Acyclic Graph
DOS	Denial Of Service
ECDHE	Elliptic Curve Diffie Hellman Ephemeral
FFNN	Feed-Forward Neural Network
GSM	Global System for Mobile Communications
IoT	Internet of Things
IETF	Internet Engineering Task Force
PSK	Pre-Shared Key
LPWA	Low Power Wide Area
M2M	Machine to Machine
MITM	Man-In-The-Middle
MQTT	Message Queue Telemetry Transport
MQTT-SN	MQTT for Sensor Network
NFC	Near-Field Communications
NIST	National Institute of Standards and Technology
OASIS	Organization for the Advancement of Structured Information Standards
OF	Objective Function
RF	Radio Frequency
RFID	Radio Frequency Identification
RPL	Routing Protocol for Low-Power and Lossy Networks
SDN	Software-Defined Networking
SSL	Secure Socket Layer
TLS	Transport Layer Security
UN	United Nations
WSNs	Wireless Sensor Networks
